# Nephrogenic fibrosing dermatopathy, cardiac calcification and pulmonary hypertension in an adolescent on chronic hemodialysis

**DOI:** 10.4103/0971-4065.42340

**Published:** 2008-04

**Authors:** J. Sharma, A. Mongia, M. Schoenaman, S. Chang, A. D'Angelo, M. Rao

**Affiliations:** Division of Pediatric Cardiology, Jamaica Hospital Medical Center, Jamaica, NY 11418, USA; 1 Division of Nephrology, The Children Hospital at Downstate/SUNY, Brooklyn, NY 11203 USA; 2 Division of Pulmonology, The Children Hospital at Downstate/SUNY, Brooklyn, NY 11203 USA

**Keywords:** Cardiac calcification, gadolinium, nephrogenic fibrosing dermatopathy

## Abstract

Nephrogenic fibrosing dermatopathy (NFD) is a systemic disorder of unknown etiology. Recent reports have associated the development of NFD with the use of gadolinium-enhanced magnetic resonance imaging (MRI). Here, we present the case of an adolescent with end-stage renal disease who died of biopsy-proven NFD and also developed cardiac calcification and clinical manifestations of pulmonary fibrosis with pulmonary hypertension. Only five cases of NFD have been reported in children, all of which were prior to the information regarding the consequences of using gadolinium. Here, we report a patient with NFD who received gadolinium while on chronic hemodialysis, 16 months prior to the onset of symptoms. Because he succumbed to this disease, we stress on the importance of eliminating the use of gadolinium-enhanced MRI examinations in children with impaired kidney function until the etiology of NFD is clarified

Nephrogenic fibrosing dermatopathy (NFD) is a recently identified sclerosing skin disorder of unknown etiology in patients with a history of renal insufficiency. Recently, Food and Drug Administration has received reports on adverse events affecting 75 patients with moderate to end-stage renal disease (ESRD) who developed NFD after receiving a gadolinium-based contrast agent for magnetic resonance imaging (MRI) studies.^1^ In fact, recent reviews of the literature imply that NFD may develop only in patients who received gadolinium-enhanced MRI examinations while suffering from severely impaired renal function.^2^,^3^

Only five pediatric cases have been reported, all prior to the information about the consequences of using gadolinium.^4^ None of these case reports address the issue of gadolinium-enhanced MRI examinations. However, further investigation in two of these patients revealed that both had received gadolinium prior to the emergence of NFD.^5^

Here, we present the case of an adolescent with ESRD who died of biopsy-proven NFD and developed cardiac calcification and clinical manifestations of pulmonary fibrosis with pulmonary hypertension. A thorough review of his clinical course revealed that he was exposed to gadolinium only once, while on chronic hemodialysis, 16 months before the onset of NFD.

## Case Report

A 14-year-old Hispanic male with ESRD due to congenital cystic renal dysplasia presented, after being on chronic hemodialysis for almost 10 years, with a 10-month history of right knee pain and a two-month history of worsening brawny hyperpigmentation of the skin of the distal extremities and painful contractures of the fingers and wrists. A 4-mm punch biopsy from the dorsum of the right foot revealed prominent dermal collagen bundles and an increased number of banal CD34/procollagen dual positive spindles (compatible with NFD).

Prior to the age of two years, the patient was on peritoneal dialysis. Between two and three years of age, he received a cadaveric kidney transplant that failed within one year. Since then, he has remained on hemodialysis three times a week. Other medical history includes renal osteodystrophy, seizure disorder and reactive airway disease. He took multiple medications, including Procardia XL, calcitriol, aspirin, erythropoietin, a Fentanyl patch, folic acid, iron, vitamins, flovent and albuterol.

**Fig. 1:**
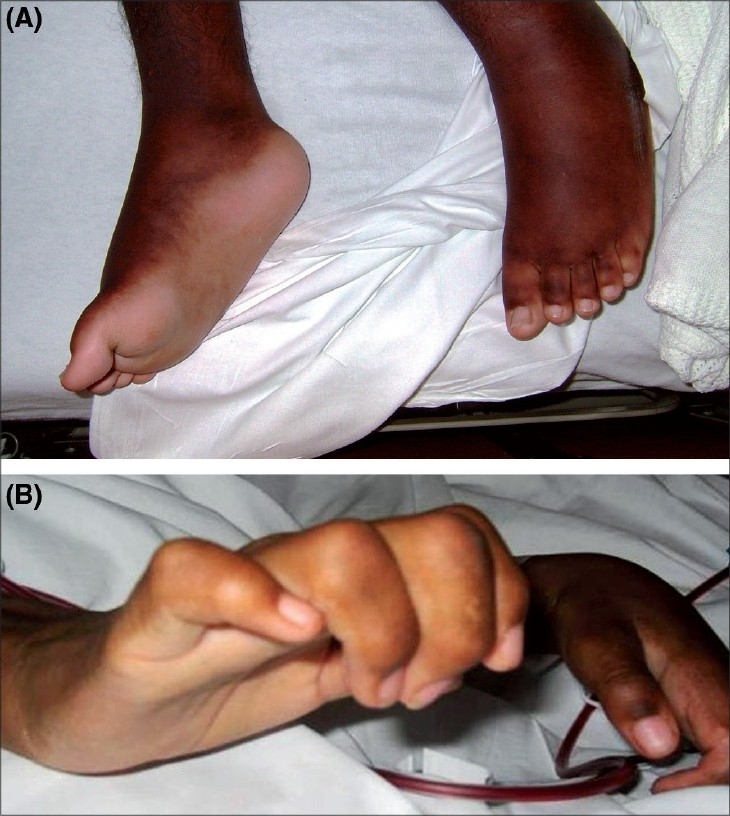
(A-B)Lesions consisting of areas of indurated plaques and nonpitting edema on the dorsal surface of the hand and foot with flexural contractures of the fingers

Physical examination showed a debilitated wheel-chair-bound adolescent with heart rate of 130 beats/min; respiration rate, 30/min and blood pressure, 140/84 mmHg. He was afebrile and hemodynamically stable, but with mild respiratory distress. Chest auscultation showed an S3 gallop, diminished air entry at the right base and bibasilar crepitations. He also had moderate hepatomegaly and ascites. Diffuse areas of thickened skin with hyperpigmentation were associated with painful contractures of the hands, knees and feet [Fig. 1].

**Fig. 2:**
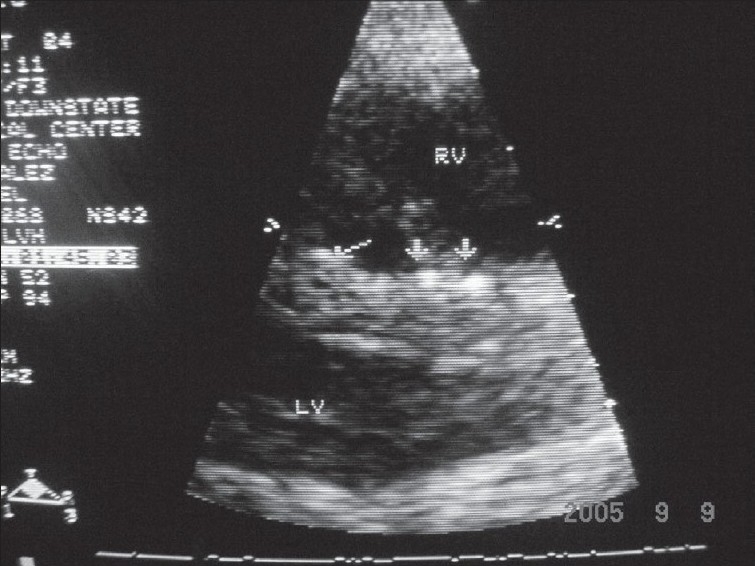
Parasternal long-axis view showing discrete small areas of calcification in interventricular septum

**Fig. 3:**
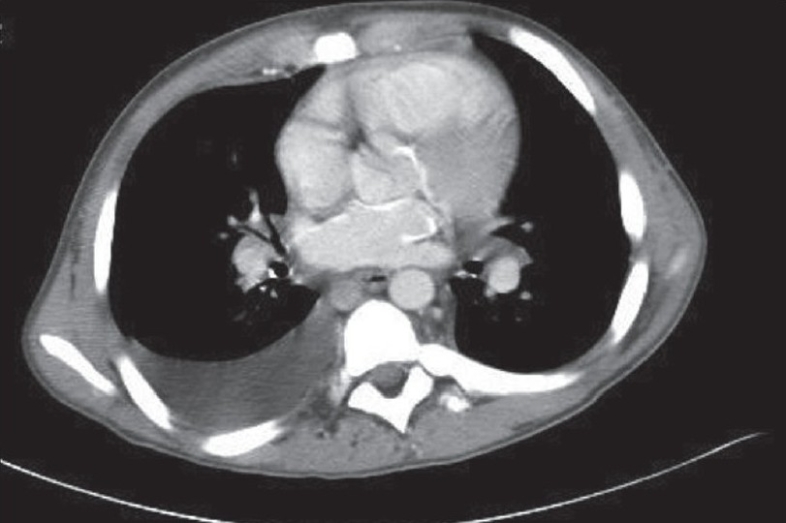
Chest CT scan showing cardiac calcification involving left atrium aortic root, proximal coronaries and mitral valve ring

Laboratory data were remarkable with erythrocyte sedimentation rate (ESR), 98 mm/h; C-reactive protein (CRP), 5.89 U; and negative serologic antibody testing for ANA, anti-smith, anti-SSA/SSB, lupus anticoagulant, anticardiolipin and anti-SCL-70. Serum intact parathyroid hormone levels and serum calcium levels over the previous 2-4 years were 580-1160 and 9-11 mg/dl, respectively. Chest x-ray demonstrated mild cardiomegaly, increased pulmonary vascular markings and a right pleural effusion. A two-dimensional-echo Doppler study demonstrated moderate concentric left ventricular hypertrophy, mild mitral regurgitation, small discrete areas of calcification in the interventricular septum and mild tricuspid regurgitation with a gradient of 64 mmHg and an estimated pulmonary artery pressure of 70 mmHg (concomitant blood pressure, 130/80 mmHg) [Fig. 2]. Chest computed tomography (CT) showed a moderate right pleural effusion, large discrete areas of pulmonary calcification and cardiac calcification involving the aortic root, proximal coronary arteries, left atrium and mitral valve ring [Fig. 3].

Within one year of the confirmed diagnosis of NFD, lung function testing showed an obstructive component with a progressive deterioration of the restrictive lung disease. It was not possible to determine whether the restriction was secondary to fibrosis, poor muscle mass, weakening muscle strength or pulmonary hypertension. Corresponding chest CT demonstrated diffuse nodules as large as 5 mm, bilateral pleural effusion and air trapping. The treatment for pulmonary vascular hypertension was initiated with sildenafil.

His condition deteriorated despite providing UVB phototherapy and physical therapy. He underwent renal transplantation, but the graft failed because of acute rejection and he returned to chronic hemodialysis. His cardio-pulmonary function steadily deteriorated and he died at the age of 16 years.

A thorough review of his previous radiology examinations revealed that, due to complications in vascular access, he had undergone a gadolinium-enhanced MRI study 16 months prior to the onset of the symptoms of NFD. He was receiving a regular three-times-a-week hemodialysis at that time.

## Discussion

NFD was first reported in 2000 by Cowper *et al.*, in 15 adult patients with a history of either hemodialysis or renal transplant.^6^ NFD typically presents with indurated skin plaques with brawny hyperpigmentation predominantly on distal extremities and trunk with sparing of the face. This skin thickening can result in contraction flexures with significant restriction in mobility. The typical histopathology of NFD closely resembles scleromyxedema except that NFD patients do not exhibit paraproteinemia or mucin deposition in other organs.^7^ Laboratory data also did not show eosinophilia and autoantibodies, as observed in other fibrosing conditions. The differential diagnosis of NFD includes scleromyxedema, scleroderma (morphea), eosinophilic fasciitis, porphyria cutanea tarda, B2 microglobulin amyloidosis, fibroblastic rheumatism, rapeseed oil ingestion and L-tryptophan ingestion.^6^

Renal failure is a common underlying risk factor for NSF/NFD. Although the specific etiological agent remains unclear, infection, auto-immunity, toxic effects of dialysate and failed kidney transplants have been considered to be the possible contributing factors.^4^,^8^ Most recently, an association with gadolinium-enhanced contrast agents used during MRI examinations in patients with moderate to severe renal insufficiency has been reported.^2^ Further, gadolinium has been detected in the tissues of renal failure patients with NFD with a history of gadolinium exposure.^9^ It is recommended that health care professionals carefully weigh the risks and benefits associated with a gadolinium-based contrast agent and, although not proven helpful, prompt dialysis after gadolinium administration is recommended to reduce the burden of this agent in the body.^2^ Our patient had undergone the study before the association between gadolinium exposure and NFD had been identified. Although he was on regular hemodialysis sessions following his first gadolinium exposure, he went on to develop the clinical features of NFD.

Management of NFD is purely empirical but includes oral steroids, photopheresis, plasmapheresis, cyclophosphamide, thalidomide, ultraviolet therapy and physical therapy.^4^,^9^–^10^ Our patient presented with typical clinical features, confirmed by histopathology on skin biopsy. The changes in NFD have been reported to be reversible with successful renal transplant. For this reason, we attempted renal transplantation even though he was highly sensitized and required multiple plasmapheresis episodes.

Patients with ESRD are at a risk of tissue calcification as a result of deranged mineral metabolism and secondary hyperparathyroidism of renal osteodystrophy. Coronary artery calcification correlates with an increased risk of cardiovascular events in adults.^11^ The usual locations for cardiac metastatic calcium deposition in patients with chronic hyperparathyroidism secondary to ESRD include AV valve/valve rings, aortic root and proximal coronaries. Our patient had a myocardial calcification involving interventricular septum as demonstrated by echocardiogram and calcification of aortic root, left atrium and proximal coronaries, as observed on CT scan.

Our patient also had significant pulmonary hypertension. Children with ESRD on chronic hemodialysis via arteriovenous access often develop unexplained pulmonary hypertension as adults.^12^ Pulmonary hypertension in such patients is usually secondary to LVH/diastolic dysfunction, uremic cardiomyopathy or recurrent thromboembolism. Pulmonary calcification and NFD are not linked to pathophysiology of pulmonary hypertension.

## Conclusion

In this study, we present the case of an adolescent with ESRD on chronic hemodialysis who developed NFD after exposure to gadolinium, complicated with myocardial calcification and pulmonary hypertension. Because of the morbidity and mortality associated with NFD, we suggest that gadolinium exposure be avoided in children with impaired kidney function, despite the ongoing hemodialysis, until the etiology of NFD is clarified.
